# Preschool Emotional Problems in the Post-Pandemic Era between Parental Risk and Protective Factors

**DOI:** 10.3390/healthcare11212862

**Published:** 2023-10-30

**Authors:** Laura Elvira Prino, Angelica Arace, Paola Zonca, Protima Agostini, Donatella Scarzello

**Affiliations:** Department of Philosophy and Education Sciences, University of Turin, 10124 Turin, Italy; angelica.arace@unito.it (A.A.); paola.zonca@unito.it (P.Z.); protima.agostini@unito.it (P.A.); donatella.scarzello@unito.it (D.S.)

**Keywords:** emotional problems, parental burnout, coping strategies, resilience, emotional competence, COVID-19

## Abstract

The psychosocial adaptation of children born or experiencing their early years during the COVID-19 pandemic remains uncertain. In order to implement prevention strategies, it is, therefore, a priority to deeply analyze children’s mental health in this post-pandemic phase and to identify family risk and protective factors. Indeed, recent studies reveal that children’s emotional distress increased with the COVID-19 pandemic, especially in situations of high parental stress. The study investigates associations between some parental characteristics (coping strategies, parental burnout, resilience, perception of social support, and promotion of children’s social-emotional competence) and children’s emotional symptoms, considering gender differences. A total of 358 parents of children aged 2 to 6 years participated in this study. Regression analyses show that parental burnout is a predictor of emotional symptoms; moreover, for females, higher levels of emotional symptoms are associated with parental maladaptive coping strategies, whereas for males, the parent’s ability to promote children’s emotional competence is a protective factor. Results emphasize the importance of supporting parental well-being as a critical factor in shielding children from the repercussions of adverse situations.

## 1. Introduction

### 1.1. The Impact of the Pandemic on Children’s Emotional Well-Being

The COVID-19 pandemic constituted a critical paranormative, that is, an unforeseen event that suddenly disrupted personal and family balances and routines. It was a potentially traumatic event, a universal introduction of risk [1] that negatively affected the mental health of both adults [2,3,4,5] and children [6,7,8,9].

With respect to children, available data from the pandemic indicate a significant increase in emotional symptoms, such as irritability, anger, anxiety, mood alterations, sleep disturbances, feeding-related issues, and hyperactivity [6,10,11,12,13,14,15].

A recent systematic review of Italian studies on children in the 0–12 age group [15] found that the children most adversely affected by the lockdown’s impact on emotional processes are those between the ages of 3 and 5 years. Preschool-aged children observed a 73.3% increase in emotional disturbances, with lower percentages observed in other age groups (35% for children under one year and 40.5% for primary school children). Preschool age also appears to be a particularly critical period for subsequent developmental trajectories and is a key target for prevention programs [15]. Indeed, mental health problems have an early onset in childhood [16,17], and longitudinal studies showed a continuous pattern of mental health sphere issues from preschool onward [18,19,20,21].

The pandemic generation (the cohort of children born during the pandemic or who experienced their first years of life under this phenomenon) has been deprived of essential relational and learning experiences due to the social isolation resulting from contagion prevention measures.

It is, therefore, essential to intervene early to identify the risk and protective factors that may affect these children’s emotional symptoms, considering the complex interaction between personal factors and experiences in the relational sphere. In terms of these relational experiences, the literature found that parents play a fundamental role in mediating the impact of a stressful event on their children’s developmental outcomes. Children’s ability to cope with a stressful and potentially traumatic event, such as a pandemic, is indeed closely interrelated with family functioning, parenting practices, and the quality of the caregiver–child relationship [10,22,23,24,25]. Children’s emotional responses to stressful events are influenced by parental reactions, and according to the spillover hypothesis, such reactions may increase children’s emotional vulnerability or, conversely, decrease it by functioning as a protective buffer [26].

Especially in the case of young children, the findings indicate that the well-being of key adults is critical to ensuring the well-being of children, so much so that supporting parents through targeted prevention programs is equivalent to investing in a “parenting vaccine” [27] meaning that it constitutes an intervention that protects children’s mental health in addition to safeguarding parental well-being. Parenting education programs can support families in daily and exceptional challenges, such as the pandemic, to interrupt the intergenerational transmission of toxic stress.

Indeed, it has long been recognized that problems in parental mental health are associated with negative outcomes in children’s mental health [28,29,30,31]. The pandemic has highlighted and intensified the already-known process of transgenerational transmission of mental disorders. The occurrence of psychopathological disorders in parents (e.g., depression and anxiety) has been found to be associated with internalizing and externalizing symptoms in their children [32,33], consistent with studies addressing the pre-pandemic period. This association is so significant, in fact, that we might borrow the title of a paper by Stracke and colleagues [33] to assert that “Mental health is a family affair” since the well-being of children is closely linked to that of parents.

Several authors have pinpointed parental stress as the key factor explaining the association between parental and children’s symptoms [24,34]. In keeping with these observations, research data show that children’s symptoms during the pandemic increased, particularly during moments of elevated parental stress levels [33,35,36]. As emphasized by attachment theory [37], children need their safe base in moments of stress, but stressed parents find it more difficult to understand their children’s needs and respond sensitively and appropriately by providing protection and reassurance. Many studies have, therefore, focused on analyzing parental stress associated with COVID-19 and its possible effects.

### 1.2. Parental Stress and Burnout

Studies on the psychological outcomes of the pandemic have shown that parents experienced high levels of stress [38,39], greater than the general population of adults [40,41].

Parents were indeed faced with multiple sources of stress during the pandemic: the tasks of providing physical care, emotional support, and financial support for children became much more difficult to cope with [42]. The lockdown and contagion-prevention measures also deprived families of support from informal networks and hampered their access to various types of services (health care, mental health, education and schooling, social services) that enable households to seek support under normal conditions; the resulting social disconnect exacerbated the burden on parents. Research has found that parents harbored numerous concerns: fears of falling ill, be it themselves, their children, or other family members; a lack of social relationships; mental health consequences; and economic and work-related problems [43,44].

Stress levels were highest among the parents of very young, preschool-aged children, probably due to their greater needs, especially during the lockdown period, considering that educational services were closed and it was not possible to take advantage of the support provided by informal networks, it fell wholly on parents to meet these needs [45,46].

Excessive parenting stress levels increased the risk of adults manifesting post-traumatic stress, anxiety, and depression [9,47,48] and practicing harsh parenting in interactions with their children; in the worst cases, they resulted in intra-familial physical and psychological violence [24,49] and the phenomenon of parental burnout [42,50,51].

Parental burnout is a chronic stress condition characterized by intense exhaustion tied to one’s parental role, leading to emotional detachment from one’s children and self-doubt about one’s parenting abilities [52]. There is a high risk of burnout when a parent perceives a significant discrepancy between what would be required to practice optimal parenting and the resources actually available to them [53].

The lockdown exacerbated the gap between the tasks required for child care and education and the resources available to meet these demands; this caused an increased risk of burnout [42,54], especially among parents with at least one young child under the age of 4 [55]. Burnout can have serious consequences on parents and the family as a whole, including in the long term [55,56]; it is therefore imperative that parental burnout risk be evaluated, and such evaluation requires a careful and systematic assessment.

### 1.3. Parental Well-Being: Coping and Protective Factors

The psychological impact of the pandemic varies considerably due to differences in the availability of resources—personal and social—both before and during the pandemic. While some families turned out to be more vulnerable, in part due to pre-existing factors (economic-social marginality, mental health problems, or family members with special needs) and limited opportunities to avail themselves of support, for many parents, the stress they experienced during the early phase of the pandemic diminished in the later phases [57,58] and did not result in lasting or clinically significant mental health problems [44]. Some families can even be said to have exhibited “post-traumatic growth” [59], that is, the ability to thrive in the face of adversity [60,61].

It is, therefore, necessary to analyze what protective factors can act as a buffer against the impact of stress on families, preventing such stress from resulting in mental health problems. Of these factors, parental emotional competence is associated with a lower risk of burnout [62]; in fact, parents with higher emotional regulation skills were better able to cope with stress during the pandemic period [63,64]. In addition, it is important to note that parental emotional competence influences how children respond to stress [65].

In addition, several studies showed that social support helps parents cope with stress [66]; its role as a protective factor for parents’ perceived stress has also been emphasized for the pandemic period [67]. These findings are consistent with studies conducted on the general population from which the protective effect of social support on mental health in stressful situations and its buffer function on the negative effects of a low level of resilience are shown [68,69].

The literature, in fact, points to the central role played by parental resilience, understood as the ability to bounce back during the pandemic [25], thereby enabling parents to provide competent and quality parenting for their children despite adverse circumstances [70,71]. Highly resilient families were able to maintain their daily routines, adapt to change, and solve problems creatively [72]. Some research data show that a high level of parental resilience protects against the risk of parental burnout [25,73] and is associated with fewer emotional symptoms in both caregivers and children [74]. Parental resilience is related to child resilience, as resilient parents are better able to foster children’s adaptation and coping skills in dealing with stressful situations [71,75,76], but there are still few studies on this issue, and the results they present are inconsistent [25].

Although many families proved resilient, as Whaley and Pfefferbaum [44] argued, the long-term effects of the pandemic are still beginning to emerge. A better understanding of risk and protective factors is therefore needed in the long term as well.

### 1.4. Current Study

The pandemic was an unprecedented event; hence, it is difficult to make predictions about the long-term effects it might have on the psychosocial adaptation of children who were born or experienced their early years during the pandemic. It is, therefore, necessary to continue to monitor such children’s well-being over time in order to identify risk and protective factors on the basis of which to design programs and services to increase their resilience to adverse events.

The first objective of this pilot study is thus to analyze the emotional symptoms of a sample of children between the ages of two and six years: the “lingering impact” of the pandemic may reveal different specificities than seen in previous phases. We also aim to analyze gender differences in emotional symptoms because the previous data are not conclusive, perhaps due in part to heterogeneity in the methodologies used and the age groups considered: some studies found that it is boys who exhibit more emotional symptoms [77,78], while others instead pointed to girls [79]. It is, therefore, useful to proceed by adopting a more gender-specific analysis of the pandemic’s impact on children.

Given that young children’s emotional well-being is closely intertwined with parental well-being and the way parents cope with stressful events while also taking into consideration the resources available to parents, the second objective of this study is to investigate the level of parental burnout, coping strategies used, and protective factors (specifically parental resilience, social support and the promotion of children’s emotional competence) while also analyzing possible associations between these aspects and parents’ socio-demographic characteristics. Available studies show that mothers suffered more negative consequences than fathers during the pandemic, exhibiting higher levels of stress and mental health problems [58,80,81], whereas the association between parental age and exhibited symptoms is unclear. According to some research, it is younger parents who present more problems [44,82], while other studies found it is older ones [9]. With respect to socioeconomic status, strain arising from financial problems increased parental stress; precarious and unfavorable economic conditions, such as low SES, may, therefore, be associated with a higher risk of both parental and child maladjustment [15,44,59].

Studies mainly focused on the association between socio-demographic characteristics and levels of stress and mental health problems, while little research investigated the specific association between parental socio-demographic characteristics, coping strategies used to deal with the pandemic, and protective factors.

As a third objective, we aim to analyze whether parental adjustment predicts children’s emotional symptoms and, if so, what dimensions of it. Many studies focused on stress, mental health problems, and parental burnout in influencing children’s emotional well-being and attempted to identify which family factors are protective for children, but to our knowledge, no research simultaneously considered the role of parental burnout, pandemic-related coping strategies, resilience, emotional competence, and social support (emotional and practical) in influencing children’s well-being, including analyzing possible differences between boys and girls.

## 2. Materials and Methods

### 2.1. Participants

Participants included 358 parents (84.9% mothers) aged 24 to 48 years old (M = 38.04; SD = 5.69), almost all of whom were Italian (92.2%). Most of the participants (64.8%) had high educational attainment (bachelor’s or postgraduate degree), 31.3% completed upper secondary school, and 3.9% completed only junior high school. Of these parents, 40.2% were employed in a white-collar profession, 11.2% were teachers, 11.7% were entrepreneurs, 5% were managers, and 5.6% were homemakers. Parents who worked exclusively from home were in the minority (7.3%). Their socioeconomic status (SES) was upper-middle level (M = 51.68; SD = 10.06), as measured by Hollingshead’s index (1975), which is a cultural and economic index based on an algorithm that combines individuals’ educational level and their profession.

Regarding marital status, most participants were married (62.1%) or cohabiting (32.9%); a limited number were single parents (2%), separated/divorced (2.5%), or widowed (0.5%).

The children for whom responses were provided (females: 50.3%) were aged between 2 and 6 years (M = 3.82; SD = 1.22); 57.8% were aged between 2 and 4 years, while 42.2% were between 4 and 6 years old.

### 2.2. Instruments 

To detect children’s emotional difficulties, we used the ***Emotional symptoms*** scale from the **Strengths and Difficulties Questionnaire** (SDQ) [83], an instrument that is employed widely to investigate strengths and weaknesses in children’s behavior. Specifically, the scale is composed of 5 items (i.e., “Nervous or clingy in new situations, easily loses confidence”) rated on a 3-point Likert scale (0: Not true, 1: Somewhat true, 2: Certainly true). Higher scores on the scale indicate that the parent filling out the questionnaire perceives that their child exhibits more behaviors relating to emotional distress. In this research, Cronbach’s α for this scale is α = 0.72.

To investigate levels of parental burnout, the **Parental Burnout Assessment** (PBA) [84] was used. This instrument consists of 23 items assessed on a 7-point Likert scale (0 = never to 6 = every day) investigating 4 dimensions of parental burnout: *Exhaustion in one’s parental role* (i.e., “I feel completely run down by my role as a parent”); *Contrast with previous parental self* (i.e., “I’m ashamed of the parent that I’ve become”); *Feelings of being fed up with one’s parental role* (i.e., “I don’t enjoy being with my child/children”); and *Emotional distancing from one’s children* (i.e., “outside the usual routines—lifts in the car, bedtime, meals—I’m no longer able to make an effort for my child/children”). In addition to the scores for the individual scales, the total score can also be used. This tool showed excellent psychometric properties in studies using it in many different countries, including Italy [85]. For our study, the reliability coefficients of the scales were: *Exhaustion in one’s parental role*: α = 0.89; *Contrast with previous parental self*: α = 0.86; *Feelings of being fed up with one’s parental role*: α = 0.81; *Emotional distancing from one’s children*: α = 0.77; and Overall score for the scale: α = 0.93.

To capture the coping strategies parents used in dealing with the pandemic, the **Robust—Pandemic Coping Scale** (R-PCS) [86] was administered. This consists of 20 items scored on a 5-point Likert scale (from 1 = never to 5 = always) and has 4 subscales, each consisting of 5 items: *Adjustment*, *Proactivity*, *Despair*, and *Aversion*. The first two measure adaptive coping strategies related to pandemics and epidemics, while the second two examine maladaptive coping strategies. In this research, the totals of adaptive and maladaptive strategies calculated as the sum of the two related subscales were also considered. The reliability coefficients of the subscales for this study are: α = 0.74 for the *Adjustment* subscale, α = 0.75 for the *Proactivity* subscale, α = 0.72 for the *Despair* subscale, and α = 0.74 for the *Aversion* subscale.

Finally, the **Parents Assessment of Protective Factors scale** (PAPF) [87] was used to analyze the existence of family protective factors. The scale consists of 36 items on a 5-point Likert scale (ranging from 0, “This is not at all like me or what I believe”, to 4, “This is very much like me or what I believe”) that assess four types of protective factors: (1) *Parental resilience*, (2) *Social connections*, (3) *Concrete support in times of need*, and (4) *Social and emotional competence of children*. The latter scale measures the parent’s ability to recognize and respond consistently to children’s emotions and promote their emotional competence. Average scores for the subscales and full measure can be interpreted as Low (0–1.99), Moderate (2.00–2.99), High (3.00–3.99), and Maximum (4.00), with higher scores representing a higher level of that protective factor. This instrument has sound psychometric properties, and the subscale reliability index (Cronbach’s α) ranges between 0.87 and 0.93 (Kiplinger & Browne, 2014). In our study, the reliability coefficients were: α = 0.88 for the *Parental resilience* subscale, α = 0.95 for the *Social connections* subscale, α = 0.89 for the *Concrete support in times of need* subscale, α = 0.93 for the *Social and emotional competence of children* subscale, and α = 0.95 for the total scale.

### 2.3. Procedure

After obtaining approval from the Ethics Committee of the home university, nurseries and preschools in the Piedmont region were contacted, and the link to the online Google Form was disseminated to families through them. The link was active from May to November 2022. All research participants gave their informed consent after receiving information about the objectives and methods of the study. Data were processed in accordance with privacy regulations.

### 2.4. Data Analysis

Data analysis was performed using the statistical package SPSS version 28.

First, descriptive analyses were carried out for all variables considered, and gender differences and associations with socio-demographic characteristics were investigated. Next, correlations between girls’ and boys’ emotional symptoms and different dimensions of parental adjustment were analyzed. Finally, several stepwise regression analyses, separate for boys and girls, were performed to investigate whether any dimensions of parental adjustment were predictors of emotional symptoms and, if so, which ones.

## 3. Results

### 3.1. Emotional Symptoms

The mean score for the sample (M = 1.68; sd = 1.84) was in line with the averages reported in the literature and indicated in studies on the Italian population as well [88]. Parents, therefore, do not detect significant problems in their children’s emotional domain. There was a slight significant difference in the mean scores obtained by girls and boys (F = 5.875; *p* < 0.05); it was the girls who scored slightly higher on this scale. Taking into consideration the age of the children, younger children scored lower on average on this scale (F = 9.279; *p* < 0.01).

### 3.2. Parental Burnout

The scores measured by the various scales of the PBA indicate low levels of parental burnout in our sample. The two subscales showing the highest scores (see Table 1) were that of *Parental role exhaustion* and *Contrast with previous parental self,* which reflect, on the one hand, the feeling that parenting requires too much commitment and that the parental role is emotionally draining and, on the other hand, the feeling of not being as good a parent as one used to be and shame around one’s parenting.

In line with previous research [89], using an analysis of variance, this study found that mothers exhibited a higher level of parental burnout than fathers, both in their total score (F = 6.59; *p* < 0.05) and in the two subscales with a higher average score in the sample, namely *Exhaustion in one’s parental role* (F = 8.05; *p* < 0.01) and *Contrast with previous parental self* (F = 6.49; *p* < 0.05). The parent’s age had a slight significant negative correlation with the total scale score (r = −0.113; *p* < 0.05) and, specifically, with the subscale *Contrast with previous parental self* (r = −0.114; *p* < 0.05). There were no associations with other socio-demographic variables, such as socioeconomic status (SES), working outside the home vs. smart working, and having one or more children. There were no differences between those who contracted COVID-19 and those who did not, even when the individual was hospitalized.

### 3.3. Coping Strategies

Consistent with previous research [86], the mean scores (Table 2) for the two challenge-focused adaptive coping dimensions, namely *Adjustment* and *Proactivity*, were higher than those for the threat-focused maladaptive coping strategies, namely *Despair* and *Aversion*. Parents who participated in the questionnaire thus perceived in the post-pandemic period that they possessed adaptive coping resources.

Differences between mothers and fathers were not significant under the analysis of variance, unlike the findings reported for previous research. Working at home or outside also did not appear to be associated with the coping strategies parents use. Age appeared to be weakly negatively correlated with the *Aversion* subscale (r = −0.114; *p* < 0.05). The *Proactivity* subscale was weakly associated with two socio-demographic characteristics of the parent: it showed positive correlations with SES (r = 0.267; *p* < 0.05) and a negative correlation with the number of children (r = −0.126; *p* < 0.019). For the *Aversion* scale, significant correlations were found in the opposite direction: with SES (r = −0.128; *p* < 0.05) and number of children (r = 0.173; *p* < 0.01). Finally, there were no differences related to having contracted COVID-19 or not and having been hospitalized due to the virus.

### 3.4. Familial Protective Factors

Administration of the instrument shows that, on average, the sample of parents participating in the research scored high on all the subscales (Table 3); only the score for the *Concrete support in times of need* scale presented an average at the upper end of “moderate” [87].

In terms of socio-demographic differences, only a few slight significant associations were found. SES correlated weakly with the *Social connections* (r = 0.117; *p* < 0.05) and *Concrete support in times of need* (r = 0.150; *p* < 0.05) subscales. Finally, having only one child was associated with higher scores on the *Children’s social and emotional competence* subscale (F = 6.63; *p* < 0.05). There were no differences associated with having contracted COVID-19 and having been hospitalized.

### 3.5. Children’s Emotional Problems and Familial Adjustment

Based on correlation analyses, the emotional symptoms of girls and boys were correlated with all scales of parental adjustment (Table 4). Emotional symptoms were positively associated with all dimensions of parental burnout and the use of maladaptive coping strategies and negatively correlated with adaptive coping strategies and all familial protective factors. As can be seen from the table, there were differences in the correlations calculated here depending on the sex of the child. The results for coping strategies and familial protective factors are worth noting. In relation to coping strategies, maladaptive strategies significantly correlated with emotional symptoms scores, especially for girls; in relation to familial protective factors, the results showed that family protective factors were more correlated with emotional symptoms for boys.

To ascertain whether parental adjustment is a predictor of the occurrence of emotional problems in children, several stepwise regression analyses were conducted. In the first analysis, the total scales for the different instruments (parental burnout, adaptive coping strategies, maladaptive coping strategies, and familial protective factors) were included as independent variables. Three different regression analyses were then carried out considering the subscales of each of the three instruments as predictors to investigate the specific dimensions of parental adjustment in more depth. To highlight possible gender differences, given the discrepancies already observed in the correlations, the analyses were carried out separately for male and female children.

Based on the first regression, for boys, emotional symptoms were predicted by parental burnout (β = 0.269; *p* < 0.001) and familial protective factors (β = −0.194; *p* < 0.05), while coping strategies were not significant predictors. For girls, parental burnout also turned out to be a predictor of emotional symptoms (β = 0.235; *p* < 0.01), but familial protective factors were not significant predictors; for female children, maladaptive coping strategies play an important role in predicting the level of emotional symptoms (β = 0.266; *p* < 0.001).

The second regression analysis focused on the dimensions of parental burnout. For boys, the *Contrast with former parental self* scale was a predictor of emotional symptoms (β = 0.354; *p* < 0.001), while for girls, it was a different aspect of burnout that predicted emotional symptoms, namely *Exhaustion in one’s parental role* (β = 0.333; *p* < 0.001).

With regard to coping strategies, in boys, *Despair* (β = 0.174; *p* < 0.05) was positively associated with emotional symptoms, while *Adjustment* was negatively associated with symptoms (β = −0.187; *p* < 0.05); for girls, both of the scales referring to maladaptive coping strategies were predictors of emotional symptoms (*Despair*: β = 0.150; *p* < 0.05; *Aversion*: β = 0.240; *p* < 0.01).

Among the family protective factors negatively associated with emotional symptoms, the role of *Social and emotional competence* is significant for boys (β = −0.352; *p* < 0.001), and that of *Parental resilience* is significant for girls (β = −0.241; *p* < 0.01).

## 4. Discussion

Although the pandemic represented a universal introduction of risk [1], the consequences were not the same for all individuals. As argued by Rosenfeld and colleagues, “the pandemic has affected everyone, but not everyone has been affected equally” [90] (p. 316); therefore, it is vital to identify which risk and protective factors explain the pandemic’s differential impact on families and children in order to implement ever more effective prevention and support measures.

This study’s first objective was to analyze, in the post-pandemic period, the prevalence of emotional symptoms in a sample of preschool children so as to also highlight possible gender differences since there are discrepancies among the data presented in the literature. Our results are consistent with studies that show a greater frequency of emotional symptoms among girls in this age group [79]. Higher scores in parent-reported emotional symptoms for daughters could also be a reflection of a differential emotional socialization process and a different focus on and interpretation of certain emotions exhibited by sons and daughters on the part of parents, which in turn reflect stereotypical gender differences and tend to reproduce culturally accepted gender roles, widespread even today, framing girls as more emotional, more prone to mood swings, more fearful, and less confident (e.g., [91,92,93,94]).

The second research objective was to analyze the level of burnout, coping strategies used, promotion of children’s emotional competence, resilience, and social support reported by parents, relating each of these aspects to the socio-demographic characteristics of the parents.

In comparison with fathers, mothers have a higher level of burnout. This result is consistent with studies showing that the negative consequences of the pandemic were more severe for mothers [58,80,81]. In contrast, no differences were found between mothers and fathers in terms of the coping strategies they used and the protective factors investigated. The higher level of maternal burnout does not depend on these aspects, therefore, but might be attributed to the unequal burden of parenting borne by mothers and fathers on a daily basis; generally, mothers are entrusted with most of the caregiving tasks. The temporary closure of schools and educational services made it particularly strenuous for them to manage their children and, in the case of working mothers, to also maintain an effective work–life balance.

Younger parents seem to be in greater distress: they have higher burnout scores and use the maladaptive coping strategy of aversion more frequently. This finding is in line with some previous studies indicating that younger parents have higher levels of stress and emotional exhaustion and increased mental health problems [44,82,95].

According to previous studies, having more than one child is a risk factor for the onset of parental burnout [95]. In our sample, in contrast, the number of children was not found to affect the level of burnout and is instead associated with coping; parents with more children are more likely to use the maladaptive coping strategy of aversion and less likely to use the adaptive coping strategy of proactivity. Furthermore, we found an association between promoting children’s emotional competence and having only one child. Having more than one child multiplies the demands children make on their parents, and thereby makes it more difficult for parents to manage stressors and use effective coping strategies. Having only one child allows more opportunities for observation and dialogue, which facilitate emotional socialization processes.

With respect to socioeconomic status, there is evidence in the literature that strain related to financial problems increases parental stress and that, in the face of the critical event of the pandemic, precarious and unfavorable economic conditions such as low SES can lead to an increased risk of maladjustment on the part of both parents and children [15,44,59]. Our results also suggest that socioeconomic status influences parental adaptation; a higher level of SES is associated with coping characterized by greater proactivity and less aversion, as well as more social, concrete, and emotional support. Contrary to findings in other studies [82] conducted in earlier periods of the pandemic, the fact of having contracted COVID-19 or not does not influence any of the variables considered. Presumably, COVID-19 has come to constitute a commonplace experience, and contracting the virus no longer qualifies as an exceptional or traumatic event capable of affecting parental adjustment.

The third and final research objective was to investigate some predictors of girls’ and boys’ emotional symptoms by considering parental burnout, pandemic-related coping strategies, resilience, promotion of children’s emotional competence, and the social support perceived.

Different predictors emerged for male and female children, with the exception of parental burnout, which was significant for both girls and boys, consistent with the many findings in the literature that show a close link between the emotional symptoms manifested by children and the level of burnout and parental stress experienced by parents [96,97]. There are no data on parental burnout in the literature that take into account gender differences in children. However, in our study analyzing the specific dimensions of burnout that predict girls’ and boys’ emotional symptoms, we did find a difference linked to the children’s gender; in the case of sons, it is the feeling of not being as good a parent as one used to be and shame around one’s parenting that is a predictor of emotional symptoms, while for daughters, it is the feeling that the parental role is emotionally draining and that parenting requires too much commitment.

The results indicate that pandemic coping strategies are a predictor of emotional symptoms only for daughters; specifically, it is the enactment of maladaptive coping strategies that predicts emotional symptoms for female children. Looking specifically at the various coping strategies, differences emerge between sons and daughters here as well; for females, both parental maladaptive strategies are significantly associated with emotional symptoms. Therefore, both Despair (i.e., feeling overwhelmed by fear, tending to fixate on the emergency, and not being able to recognize solutions and avenues for coping with problems) and Aversion (i.e., the degree of rejecting the public health protection measures established by applicable authorities, perceiving such rules as a threat to the need for autonomy) are significant. For boys, in contrast, the significant aspects are one parental maladaptive strategy (Despair) and one parental adaptive strategy (Adaptation), each of which has the opposite effect on emotional issues. Analyzing which coping strategies parents use in relation to the pandemic is highly important. According to the family stress model, environmental stressors, such as those related to the pandemic situation (e.g., social isolation, difficult balancing work time and family time due to smart working, financial difficulties, risk of job loss, trouble managing homeschooling, and increased parenting duties) interfere with family dynamics, can negatively affect parenting [98,99,100]. Due to a spillover effect, parental stress leads to intensified negative interactions between parents and children and, as a cascade, an increased risk of negative outcomes in children [100]. Individuals respond to stress differently depending on their subjective perception of the situation and the coping strategies they employ [101,102]. Parents who are able to enact functional coping strategies in dealing with stressors may become positive role models from whom children learn by observation, thus acquiring their own functional coping strategies for dealing with stress. The differing effects observed on male and female children are worth investigating further, with a view to calibrating parenting support efforts to children’s characteristics so as to make them more effective.

Finally, family protective factors are negatively associated with emotional symptoms in boys and not in girls in the analysis that considered the broader dimensions of the constructs investigated. Looking in detail at the various family protective factors, parental resilience, namely the process of coping with stress and functioning well in the face of stressors, challenges, or adversity, was significant for females. The result of parental resilience is personal growth and positive change. This finding is in line with the literature on resilience, in which it is shown to play a key role as a protective factor for child development [70,103].

For boys, on the other hand, the family protective factor associated with decreased emotional symptoms is the parental ability to promote children’s social and emotional competence. Children’s emotional competence, in fact, is developed through an environment and experiences that allow them to establish close and secure relationships with adults and peers and to experience, regulate, and express their emotions. The fostering of this dimension requires the parent to have a strong degree of social and emotional competence. Indeed, the literature points out that parents must have emotional competence in order to foster this quality in their children [104,105]. Children learn to understand, express, and regulate emotions thanks to parental support [106,107], and such support is especially important in the early years of life [108,109], during which the caregiver acts as an external regulator of the child’s emotions [110]. Our results are thus consistent with a number of studies indicating that parents who have enacted “emotion coaching” processes in relation to their children’s negative emotions were better able to buffer the effects of stress on children and that, as a result, such children exhibited fewer symptoms [111]. In addition, the findings agree with studies showing that, at preschool age, boys, more so than girls, are in need of nurturing environments in which sensitive caregivers act as external regulators of their emotions [112,113].

### Limitations of the Study

The study has some limitations. First of all, as a cross-sectional study, it does not allow for the identification of cause-and-effect relationships; a possible bidirectionality of influence between the variables must also be considered possible. For example, children’s emotional symptoms could increase parental burnout: in fact, some studies show that children’s emotional dysregulation predicts parental stress [114], and so, as suggested by Johnson and colleagues [57], one must be cautious in positing a one-sided relationship. Second, the questionnaire link was disseminated through nurseries and preschools in the area; the participants are thus residents of a region in northern Italy, which means that the results cannot be generalized to the Italian population as a whole, also because most respondents have medium or high SES and there are few fathers in the sample. The fact that participation was voluntary probably favored the participation of parents who were attentive and sensitive to the issues at hand and familiar with computers and other electronic devices. The difficulty in involving representative samples of the general population is common to many studies on this topic, as reported in several systematic reviews [9,15,75]. It would be interesting to replicate the study on a more heterogeneous sample in terms of socio-demographic characteristics that also include high-risk families so as to test the link between the constructs investigated and the occurrence of clinically significant symptomatology, aspects that could not be explored in our study given the small number of children with high emotional symptom scores and parents with high levels of parental burnout, prone to dysfunctional coping strategies and possessing few protective factors. Finally, it would also be interesting to take into account the assessment of children’s emotional symptoms by an independent observer to be compared with the parent’s evaluation.

## 5. Conclusions

Our study shows that in order to counteract the risk of negative effects on the emotional development of this “pandemic generation” of children, it is essential to invest in parental well-being that can serve as a kind of parenting vaccine against the repercussions of adverse events. In particular, according to our data, parenting support programs should aim to strengthen the ability to manage stress and the use of adaptive coping strategies. Another important dimension of family intervention is to promote parents’ emotional competence and their ability to recognize children’s needs and respond to them sensitively by promoting their emotional competence through a cascading effect.

Consistent with currently available studies on the psychological effects of the pandemic, the analysis of our data led to the conclusion that a range of risk and protective factors need to be considered—there is no single dominant factor that predicts outcomes [44]—and further research in this area is needed in order to field interventions aimed at protecting the mental health of boys, girls, and families so as to foster their resilience in the face of highly stressful situations, such as the one we have just experienced, that might arise in the future.

## Figures and Tables

**Table 1:** Parental Burnout Assessment (PBA) descriptive statistics.

	Min	Max	Mean	S.D.
Exhaustion in one’s parental role	0.00	5.00	0.872	0.949
Contrast with previous parental self	0.00	4.67	0.449	0.726
Feelings of being fed up with one’s parental role	0.00	4.60	0.248	0.518
Emotional distancing from one’s children	0.00	5.67	0.323	0.663
Total scale	0.00	4.11	0.473	0.624

**Table 2:** Robust—Pandemic Coping Scale (R-PCS) descriptive statistics.

	Min	Max	Mean	S.D.
Adjustment	1.00	5.00	3.64	0.75
Proactivity	1.00	5.00	4.05	0.69
Despair	1.00	4.60	1.94	0.72
Aversion	1.00	5.00	2.11	0.73
Adaptive coping strategies	2.00	10.00	7.68	1.26
Maladaptive coping strategies	2.00	8.80	4.06	1.12

**Table 3:** Parents Assessment of Protective Factors scale (PAPF) descriptive statistics.

	Min	Max	Mean	S.D.
Parental resilience	0.00	4.00	3.31	0.56
Social connections	0.00	4.00	3.05	0.92
Concrete support in times of need	0.11	4.00	2.99	0.73
Social and emotional competence of children	0.00	4.00	3.06	0.67
Total scale	0.11	16.00	12.41	2.32

**Table 4:** Correlations between sons’ and daughters’ emotional symptoms and parental adjustment.

		Emotional Symptoms Scale (SDQ)		
		Total Sample	M	F
PBA	Exhaustion in one’s parental role	0.301 **	0.298 **	0.333 **
	Contrast with previous parental self	0.292 **	0.354 **	0.251 **
	Feelings of being fed up with one’s parental role	0.210 **	0.284 **	0.166 *
	Emotional distancing from one’s children	0.221 **	0.278 **	0.180 *
	Total scale	0.301 **	0.348 **	0.281 **
R-PCS	Adjustment	−0.183 **	−0.215 **	−0.174 *
	Proactivity	−0.123 *	n.s.	−0.151 *
	Despair	0.195 **	0.204 **	0.207 **
	Aversion	0.183 **	n.s.	0.275 **
	Adaptive coping strategies	−0.176 **	−0.186 *	−0.187 *
	Maladaptive coping strategies	0.246 **	0.182 *	0.307 **
PAPF	Parental resilience	−0.280 **	−0.348 **	−0.241 **
	Social connections	−0.124 *	−0.168 *	n.s.
	Concrete support in times of need	−0.168 **	−0.187 *	−0.157 *
	Social and emotional competence of children	−0.236 **	−0.352 **	−0.155 *
	Total scale	−0.237 **	−0.303 **	−0.199 **

** *p* < 0.01, * *p* < 0.05.

## Data Availability

The data presented in this study are available on request from the corresponding author.
